# Biodegradable Chitosan Decreases the Immune Response to *Trichinella spiralis* in Mice

**DOI:** 10.3390/molecules22112008

**Published:** 2017-11-18

**Authors:** Klaudia Brodaczewska, Natalia Wolaniuk, Katarzyna Lewandowska, Katarzyna Donskow-Łysoniewska, Maria Doligalska

**Affiliations:** 1 Department of Parasitology, Faculty of Biology, University of Warsaw, Miecznikowa 1, 02-096 Warsaw, Poland; klaudiab@biol.uw.edu.pl (K.B.); natalawolaniuk@wp.pl (N.W.); zuzia@biol.uw.edu.pl (K.D.-Ł.); 2 Faculty of Chemistry, Nicolaus Copernicus University in Toruń, 87-100 Toruń, Poland; reol@chem.umk.pl

**Keywords:** chitosan, *Trichinella spiralis*, myeloid cells, immunosuppression, peritoneal IgA

## Abstract

The purpose of this study was to evaluate the potential of chitosan units released during natural degradation of the polymer to activate the immune system against *T. spiralis* infection. High molecular weight chitosan was injected intraperitoneally into C57BL/6 mice. Flow cytometry and cytokine concentration, measured by ELISA, were used to characterize peritoneal cell populations during *T. spiralis* infection. The strong chemo-attractive properties of chitosan caused considerable infiltration into the peritoneal cavity of CD11b^+^ cells, with reduced expression of MHC class II, CD80, CD86, Dectin-1 or CD23 receptors in comparison to *T. spiralis*-infected mice. After prolonged chitosan biodegradation, cell populations expressing IL-4R, MR and Dectin-1 receptors were found to coexist with elevated IL-6, IL-10, TGF-β and IgA production. IgA cross-reacted with *T. spiralis* antigen and chitosan. It was found that chitosan treatment attracted immune cells with low activity, which resulted in the number of nematodes increasing. The glucosamine and *N*-acetyl-D-glucosamine residues were recognized by wheat germ agglutinin (WGA) lectin and therefore any biodegradable chitosan units may actively downregulate the immune response to the parasite. The findings are relevant for both people and animals treated with chitosan preparations.

## 1. Introduction

There are many proposals for the use of polysaccharide biomaterials in medicine. Due to their physical and chemical properties, many of these substances are suitable for therapeutic purposes [1,2]. However, before these compounds can find full use as drug carriers, implants or regenerative materials in dressings, their immunogenic properties need to be clearly identified and characterized [3]. Polysaccharides are recognized by distinct cell receptors, and may induce or modulate the immune response, depending on their size and structure [4,5,6].

Chitosan and its derivatives are believed to be non-toxic, bioactive, biocompatible and biodegradable substances with low immunogenicity [7,8]. The polymer acts as a temporary extracellular matrix; it is endocytosed by cells and eventually broken down by lysozyme, after which chitosan chains with low degrees of acetylation may remain active in the body for several months [9]. Chitosan is soluble in dilute acids, and the number fraction of *N*-acetylglucosamine (GlcNAc) residues in the polymer chain, designated by the degree of acetylation, influences cell response by modulating the solubility, reactivity, biodegradability and activity of the polymer [10,11]. During biological degradation, the length of the released chains will influence the degree to which chitosan may differentially affect the outcome of the immune response. The polymer is gradually degraded, and its distinct surface activity or shared moieties may affect receptor cell signaling during immune activation. The positively charged d-glucosamine (GlcN) units may electrostatically interact with the negatively charged cell surface, and the polymer may slightly destabilize the plasma membrane [12]. It has been suggested that chitosan enhances both humoral and cell-mediated immune responses following antigen vaccination [13].

To better understand the mode by which chitosan influences the immune system, more research is needed into the immune reactivity induced by the primary structure of the polymer decomposing during its biodegradation. Therefore, the present study evaluates the activity of chitosan in a complex system consisting of a long-term parasitic infection in laboratory mice, with the level of *T. spiralis* infection being used as an indicator of the potential impact of chitosan-derived units on the immune response.

*Trichinella spiralis* infects many host species including rodents, pigs and humans. Human trichinellosis has been documented in 55 (27.8%) countries around the world [14]. Endemic outbreaks of trichinellosis not only have serious veterinary and medical implications, but also prevent the production of safe, high-quality food.

Infection with the nematode begins with the consumption of meat containing encysted larvae. Over the course of 30 h, four molts are completed, with the larvae growing and developing into adults. The inflammatory reactions induced by the nematodes result in the destruction and altered function of enterocytes in the host intestine. Newborn larvae released by the females into the lymphatic sinuses migrate via the blood and lymphatic vessels into the muscles. The larvae settle in myotubes, where they are encapsulated into nurse cells. All these nematode stages act as sources of a plethora of signals detected by cell receptors on the columnar epithelium in the intestine and by cells of the innate immune system. An effective immune response results in parasite expulsion from the intestine and a reduction in the number of muscle larvae. Myeloid, rather than lymphoid, cells are involved in the expulsion [15,16]. Secretory antigens excreted by the larvae suppress inflammation by modulating parasite-specific immune responses. *T. spiralis* and its products induce Th2- and Th3-type responses [17].

The intensity of inflammation seems to play a pivotal role in the early phase of infection when creating a suitable environment in the intestine for the parasite [18]. Carbohydrate residues are a prime target for immune recognition through the action of glycan-binding host proteins [19]. Glycoproteins have been implicated in the stimulation or evasion of host immunity: pathogens subvert the host defenses by interfering with molecules involved in inflammatory signaling [20]. The modification of protein antigens by glycans may change cellular uptake, proteolytic processing, presentation by MHC molecules and subsequent T-cell priming [21]. The present study uses chitosan, a deacetylated polymeric derivative of chitin, as a source of GlcNAc and glucosamine (GlcN) chains for the activation of cells in the peritoneal cavity. The amino sugar, GlcNAc plays an important role in cell signaling by the glycosylation of proteins [22]. The molecule is highly represented in *T. spiralis N*-glycans, and is typically found in the terminal position, or combined with *N*-acetylgalactosamine (GalNAc) in the carbohydrate chain (“LacdiNAC”: GalNAc-b1,4-GlcNAC). Interestingly, only the intestinal and muscular larval stages of *T. spiralis* synthesize a glycoprotein which specifically binds to a form of lectin known as wheat germ agglutinin (WGA) [23,24]. The structure of carbohydrates is crucial for their interaction with receptors during cell signaling and may induce immunosuppression [25]. As a biodegradable material, chitosan is well suited for evaluating the significance of glycans in the immune response; it is a potent source of high and low molecular-weight polymer chains or oligochains, which may allow functional immunoregulation supporting tissue regeneration [4]. In addition, the biological function of biodegraded chitosan units in immune regulation during parasitic infection needs to be better understood.

The aim of our studies was to evaluate the immune properties of naturally biodegraded chitosan units, which act as a model for the education of the immune system, and may determine their relevance in *T. spiralis* infection in mice.

## 2. Results

### 2.1. Changes in the Peritoneal Cell Population

**Figure 1:**
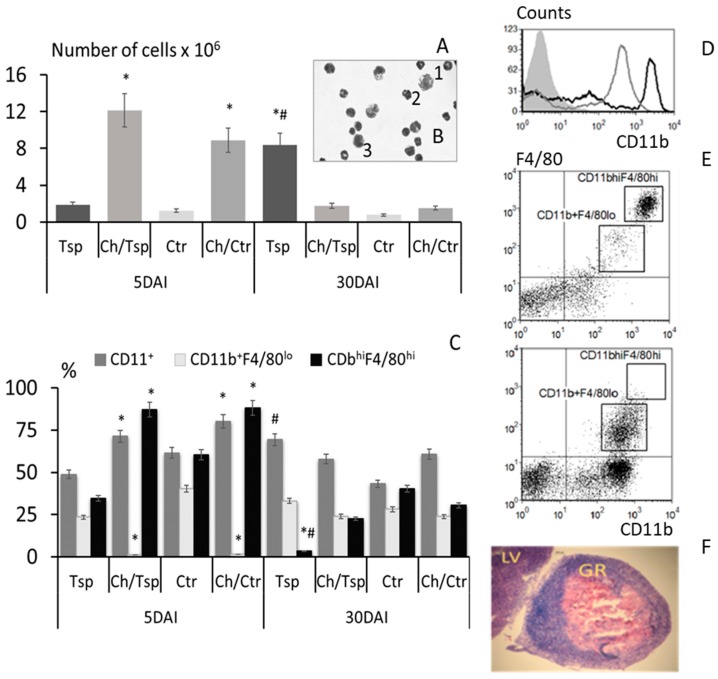
The cell response in the peritoneal cavity of mice injected intraperitoneally with chitosan and infected with *T. spiralis* five and 30 days after infection (DAI): (**A**) the number of cells; (**B**) the peritoneal cell glass smear from mice injected with chitosan then infected with *T. spiralis* (the smear being taken five days after infection). Giemsa stain; **B**1: macrophage, **B**2: neutrophil, **B**3: monocyte; the percentage of myeloid cell populations: (**C**) CD11b^+^ cells; CD11b^+^F4/80^hi^—resident macrophages; CD11b^+^F4/80^lo^—monocytes; (**D**) the number of cells identified by FACS: grey area—unstained cells, black line—after infection with *T. spiralis*; grey line—after injection with chitosan and *T. spiralis* infection on day 5; (**E**) representative dot plot analysis of cell populations in mice on day 5 following *T. spiralis* infection (**upper**); and in those injected with chitosan (**lower**); (**F**) hematoxylin stained cross section of liver (LV) with chitosan filled granuloma (GR). (*) *p* < 0.05 chitosan vs. non treated; (^#^) *p* < 0.05 infected vs. control.

The number of cells increased nine-fold in mice infected with *T. spiralis* and injected with chitosan (Figure 1A,D).

The peritoneal cell suspension in the glass smear showed that macrophages and monocytes were present but neutrophils dominated (Figure 1B). Thirty days post infection, the number of these cells significantly increased in mice infected with *T. spiralis* but dropped in mice treated with chitosan.

An increase in the percentage of CD11b^+^ cells in the peritoneal cavity was observed in the enteral phase of infection (Figure 1C). The pattern of response changed on day 30 of the experiment, and the highest percentage of CD11b^+^ cells appeared in mice infected with *T. spiralis*, with the number being slightly lower in the mice injected with chitosan.

Resident macrophages and migrating monocytes were recognized based on the level of CD11b^+^ and F4/80 receptor expression as CD11b^hi^F4/80^hi^ and CD11b^+^F4/80^lo^, respectively (Figure 1C,E). The number of resident macrophages fell significantly only in groups injected with chitosan in the enteral phase of infection. The percentage of monocytes, CD11b^+^F4/80^lo^ cells, fell in mice infected with *T. spiralis*, but still constituted over 85% of the CD11b^+^ cell population in mice given chitosan (Figure 1C). In all groups of infected and/or treated mice, the percentage of monocytes dropped during the muscle phase of *T. spiralis* infection in comparison to untreated and uninfected mice. Chitosan significantly affected the percentage of myeloid cells in the peritoneal cavity and strongly attracted CD11b^+^ monocytes into the peritoneum. Chitosan induced chronic peritonitis, accompanied by the precipitation and adhesion of chitosan onto the mesentery epithelium with granuloma formation on the surface of the liver (Figure 1F).

Five days after infection, the number of cells in the peritoneal cavity was lower in mice infected with the nematode than uninfected mice. The response of peritoneal cells was enhanced 30 days after infection with domination of resident macrophages.

### 2.2. Changes in the Percentage of Cells Expressing Immune Receptors

**Figure 2:**
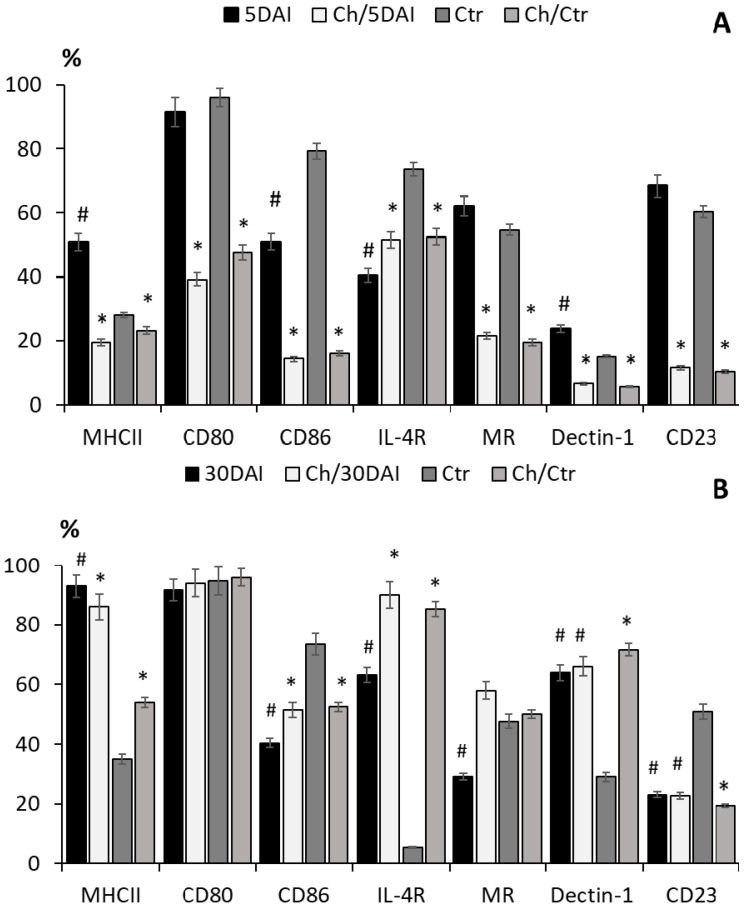

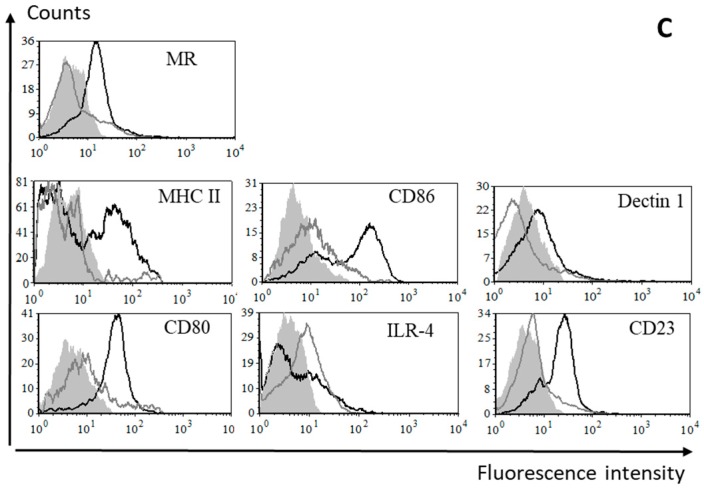
The level of receptor expression on CD11b^+^ cells in the peritoneal cavity in mice infected with *T. spiralis* and/or injected with chitosan 5 days after infection (DAI) (**A**) and 30 DAI (**B**); representative dot plots 5 DAI (**C**): grey area—unlabeled cells, black line—*T. spiralis* infected mice, grey line—treated with chitosan and infected with the nematode. (*) *p* < 0.05, chitosan vs. non treated; (^#^) *p* < 0.05, infected vs. control.

The percentage of CD11b^+^ monocytes in the peritoneal cavity expressing surface immune markers was modified differently by *T. spiralis* and by chitosan (Figure 2A–C).

Five days post *T. spiralis* infection, the percentage of cells expressing MHC class II, MR, Dectin-1 or CD23 increased in untreated mice, while the percentage of cells with MHC class II, CD80, CD86, Dectin-1 or CD23 expression dramatically fell in mice treated with chitosan. Thirty days post infection, the cell populations expressing MHC class II, IL-4R and Dectin-1 were increased, but those expressing the costimulatory receptor CD86, and MR or CD23 were reduced. After a prolonged period of biodegradation, chitosan increased the proportions of IL-4R^+^, MR^+^ and Dectin-1^+^ cells at 30 DPI.

After chitosan treatment, the percentage of cells demonstrating enhanced receptor expression fell during the intestinal phase of *T. spiralis* infection; however, chitosan supported IL-4R and Dectin-1 related cell activation in the muscle phase.

### 2.3. The Level of Cytokines in the Peritoneal Fluid

**Figure 3:**
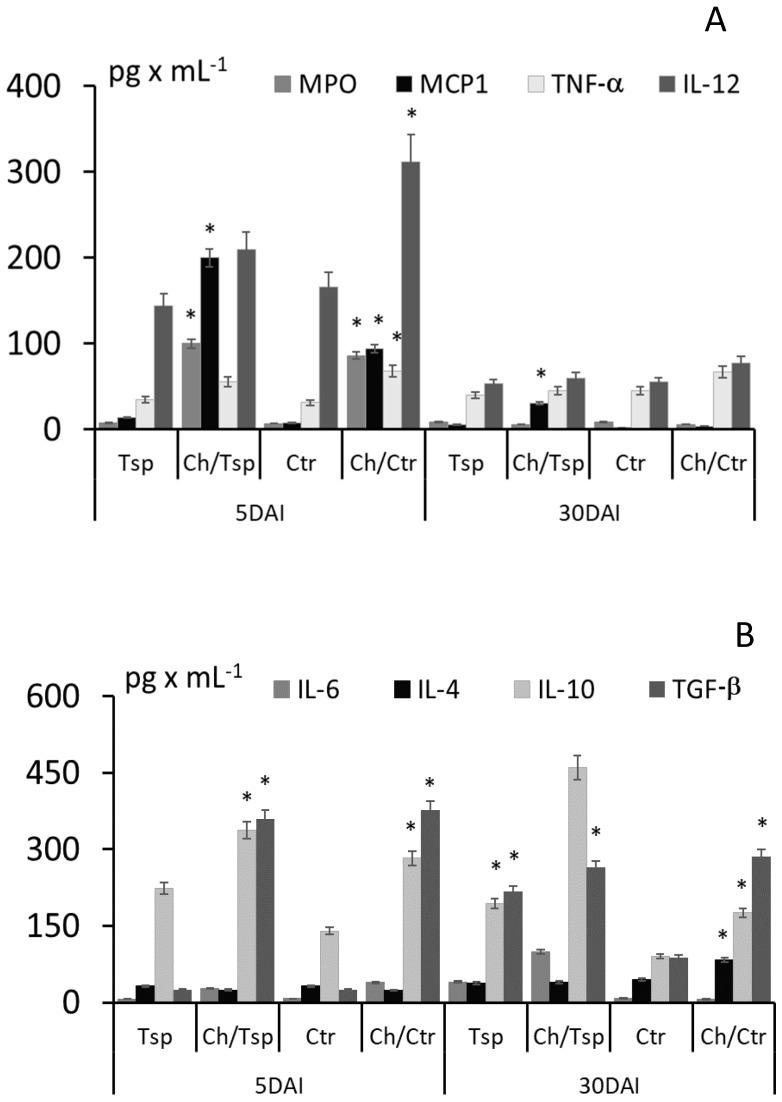
Myeloperoxidase (MPO) and cytokine response **A**: MCP-1, TNF-α; and **B**: IL-6, IL-4, IL-10, TGF-β in the peritoneal fluid of mice injected with chitosan and infected with *T. spiralis*: 5 and 30 DAI. (*) *p* < 0.05, all groups vs. control, uninfected. In the first period of treatment, chitosan supported inflammation and the response became a regulatory milieu.

Five days post infection with *T. spiralis*, chitosan appeared to influence the levels of myeloperoxidase (MPO) and cytokines in mice: higher levels of MPO, MCP-1, TNF-α, IL-12p70, IL-6, IL-10 and TGF-β were found in infected mice treated with chitosan compared to those who were not. Thirty days post infection, chitosan treatment resulted in MPO, MCP-1, TNF-α, IL-12p70 and IL-4 levels falling to control values, but IL-6, IL-10 and TGF-β were elevated (Figure 3).

### 2.4. IgA Response Specific to Muscle Larvae Somatic Antigen

**Figure 4:**
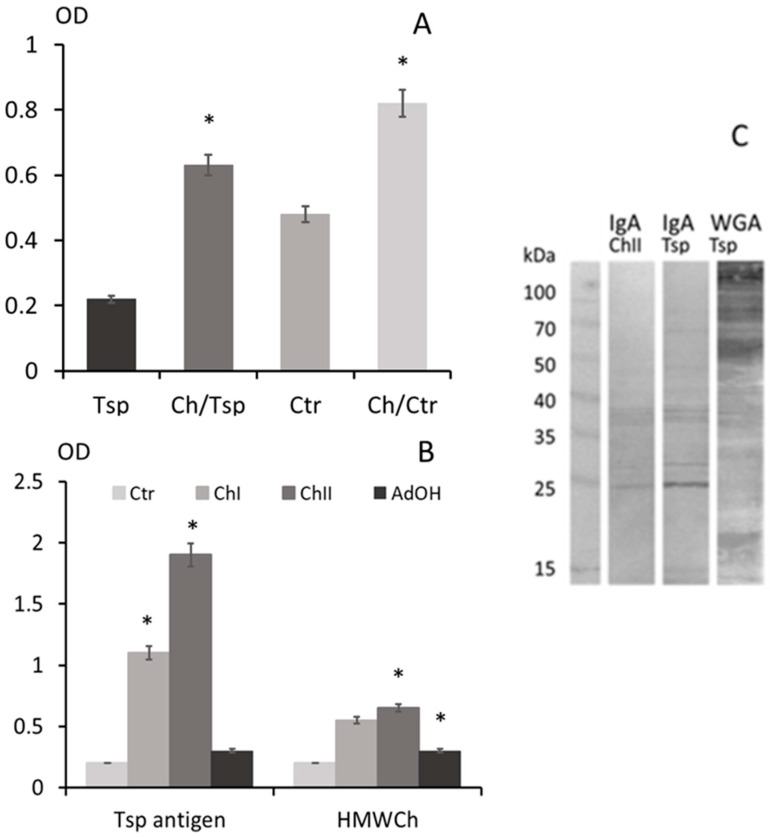
IgA response in the peritoneal cavity of *T. spiralis* infected mice, either with or without chitosan treatment; the level of IgA specific to muscle larvae somatic antigen identified by ELISA (**A**); the IgA of mice only injected with chitosan recognized *T. spiralis* larvae antigen and high molecular weight chitosan on a ELISA plate (**B**); Western-blot analysis showed that peritoneal IgA from mice injected with chitosan (ChII) and from mice infected with *T. spiralis* (Tsp) recognized similar glycoprotein bands on somatic larvae antigen at 30 DAI; WGA recognized GlcNAc residues between 50 kDa and 35 kDa. (*) *p* < 0.05 chitosan vs. non treated. The IgA induced by chitosan cross-reacted with *T. spiralis* muscle larvae antigens.

In untreated infected mice, the level of peritoneal IgA specific to *T. spiralis* muscle larvae antigens was around 2.5 times greater at 30 days after infection (DAI) than at 5 DAI. However, the IgA levels at both 5 DAI and 30 DAI were higher in the mice injected with chitosan (Figure 4A).

Western blot analysis revealed IgA cross-reactivity to muscle larvae somatic proteins of non-infected mice injected with high molecular weight (HMW) chitosan (Ch) only, and of mice infected with *T. spiralis* at 30 DAI. The IgA of mice injected with chitosan and the IgA of *T. spiralis* infected mice recognized 12 bands of *T. spiralis* antigen with the following molecular weights: 65, 52, 40, 38, 33.6, 30.6, 27, 24.6, 21.5, 20, 17.9 and 15 kDa. WGA lectin recognized the GlcNAc residues on seven bands of nematode glycoproteins with relative molecular weights 66.3, 40.3, 38.3, 23.5, 21.2, 17.2 and 15.3 kDa (Figure 4C).

### 2.5. The Level of T. spiralis Infection in Mice Injected with Chitosan

**Figure 5:**
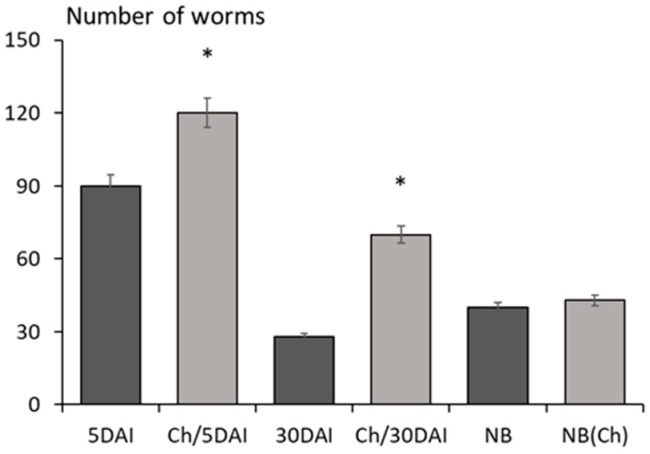
The number of adult forms in the intestine (5 DAI) and larvae × 10^2^/g of mice muscle tissue (30 DAI) in mice infected with *T. spiralis*; NB: newborn larvae delivered by females isolated from control infection; NB(Ch): newborn larvae delivered by females isolated from mice infected and treated with chitosan. (*) *p* < 0.05, chitosan vs. infected.

The numbers of adult forms and muscle larvae were greater in mice injected with chitosan than in control infection; however, the number of newborn larvae was unchanged (Figure 5).

### 2.6. Biodegradation of Chitosan Injected into the Peritoneal Cavity

**Figure 6:**
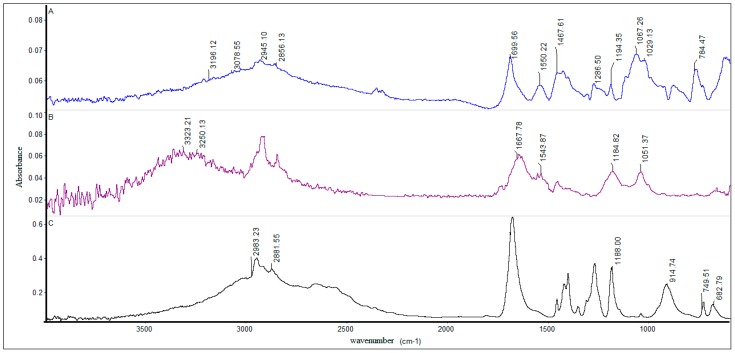
FTIR spectra of HMW chitosan: (**A**) dissolved in adipic acid; (**B**) isolated from the peritoneal cavity ten days after chitosan injection; (**C**) isolated from precipitated material in the granuloma 35 days after chitosan injection.

Attenuated total reflectance (ATR) analysis was performed. The FTIR spectra of the chitosan contained bands typical of chitosan as a salt: 3365 cm^−1^ (amide), 3292 cm^−1^ (N–H stretching), 2878 cm^−1^ (C–H stretching, present also in adipic acid), 1640 cm^−1^ (amide I), 1563 cm^−1^ (amide II), 1550 cm^−1^ (NH_3_^+^), 1155 cm^−1^ (O–C–O stretching), 1100–1020 cm^−1^ (C–O stretching of: C–OH, C–O–C cyclic, CH_2_OH groups) as shown in Figure 6, spectrum A. The analysis of peritoneal fluids confirmed the presence of chitosan; however, the spectra were slightly changed. The single peak initially present at 1700 cm^−1^ had been spread to the range 1690–1550 cm^−1^, and a new band at 1742 cm^−1^ was observed, suggesting the presence of new free carboxyl groups (Figure 6, spectrum B).

The FTIR spectrum of the sample isolated from precipitated material in the granuloma 35 days after chitosan injection exhibited characteristic bands only for adipic acid (Figure 6, spectrum C) [26]. The chitosan injected into the peritoneal cavity had biodegraded.

## 3. Discussion

The present study examines the role played by biodegradable chitosan particles in the modification of the immune response to *T. spiralis* infection. As noted previously in a different model [27], chitosan is believed to induce chronic peritonitis, accompanied by the precipitation and adhesion of polysaccharide onto the mesentery epithelium with granuloma formation at the surface of the liver. The strong chemo-attractive properties of chitosan resulted in a huge number of cells infiltrating the peritoneal cavity.

In mice injected with chitosan, the number of cells in the peritoneum dramatically increased and the proportion of CD11b^hi^F4/80^hi^ cells of the total CD11b^+^ population was depleted. The large resident macrophage population was mostly replaced by another myeloid cell population with the CD11b^+^F4/80^lo^ phenotype of a nonresponsive state and a non-resident cell phenotype [28,29]. In mice infected with *T. spiralis*, the percentage of CD11b^+^ cells expressing MHC class II molecules was found to be greater, and these cells might be derived from blood monocytes which had entered the peritoneal cavity after stimulation with parasitic antigens [30,31].

In the muscle phase of *T. spiralis* infection, the regulatory cell phenotype was favored, and the percentage of the CD11b^+^ cell population co-expressing IL-4R and MR indicated the presence of alternatively activated macrophages [32]. The macrophage marker F4/80 is also expressed at low levels on monocytes [33]. The cells were characterized as not fully activated, and the percentage expressing MHC class II, CD80, CD86, IL-4R, MR, Dectin-1 and CD23 receptors was lower. The number of myeloid cells and the level of myeloperoxidase remained temporarily elevated, but then fell as the chitosan precipitated [34].

During *T. spiralis* infection, a mixed Th1/Th2 immune response develops [35]. A skewed immune response profile can be seen following chitosan treatment, reflected in an increase in MCP-1 and IL-12p70 production, typical for Th1-induced responses; a rather weak IL-4 response was also observed, together with greatly elevated IL-6 and IL-10 levels. The clear increase in TGF-β and IL-10 observed after chitosan treatment is typical of a regulatory response [36,37,38]. TGF-β is known to be essential for parasite survival [39], *T. spiralis* stages induce the production of mediators that increase Treg cell numbers in the host [40]. In addition, native *T. spiralis* excretory-secretory products have been found to stimulate increased IL-10 and TGF-β production by dendritic cells (DCs) [41]. Chitosan treatment also greatly increased TGF-β and IL-10 production, and a greater number of the nematodes survived.

Chitosan administered into the peritoneal cavity was biodegraded, which acted as a source of polymer chains with a distinct molecular weight; this could skew the Th2 response into a Treg response following recognition by DCs [42]. The structural analysis of the chitosan in the peritoneal cavity, confirmed by the examination of nuclear magnetic resonance difference spectra, indicated the presence of chemical functional groups; the findings may be considered as the spectrum of the degradation products and adipic acid residue [26]. In addition, the presence of new COOH groups confirmed the degradation of chitosan into shorter chains [43,44,45].

Chitosan strongly attracted neutrophils [46]. The glass smears revealed the presence of numerous neutrophils in the peritoneal fluid until day 10 following chitosan treatment. These cells are able to recognize chitosan even without injury being present, and in vivo chemotaxis of neutrophils to biodegradable chitosan continues until all particles are completely internalized inside phagocytes [47]. In inflammatory disorders, neutrophils are the main source of chitinases [48]. During endocytosis, chitosan would be biodegraded into its primary structural units such carbohydrate residues [49], and this is important for the dynamics of the immune response. Chitosan clearance is also accompanied by mesenchymal stem cell recruitment and by granuloma tissue formation [47], which was also observed in our studies.

The exact mechanisms that mediate the degradation of chitosan in vivo remain poorly defined. The polymer prepared by enzymatic hydrolysis and analyzed by HPLC revealed that the chitosan oligosaccharide mixture contained oligosaccharides with different degrees of polymerization [49]. The stimulatory effects of chitosan oligosaccharide on macrophages are mediated in a size-dependent and pathway-specific manner, and although the present study provides no direct evidence that the enzymes released in vivo directly degraded the polymer before endocytosis, our findings nevertheless suggest that HMW chitosan was degraded in the peritoneal cavity, as demonstrated by the changes observed in the FTRI profile spectra. Particles of the chitosan mixture would be phagocytosed by cells. This aspect of our study requires further evaluation, especially the kinetics regarding the degree of depolymerization of the high molecular weight chitosan particles deposited into the peritoneal cavity. In addition, the incoming macrophages may persist around chitosan materials until the compound is completely cleared [50]. The chitosan attracted macrophages, and induced cell migration and proliferation at the site of the destroyed tissue, as also noted in previous studies [51,52].

The binding specificities of GlcN and/or GlcNAc residues of chains released by chitosan biodegradation were identified by WGA lectin bind assays. These may play a crucial role in the outcome of this response, probably by elevating the levels of the Dectin-1 receptor [53]. The inflammatory response of the cells could be abrogated by the overloading of GlcN or GlcNAc [54,55]. The peritoneal cells of the studied mice demonstrated low expression of the immune receptors essential for antigen recognition and presentation, and the immature myelocytes newly attracted into the peritoneal cavity were characterized by weak immune activation [56]. The activation of T cells without co-stimulation may lead to T cell anergy, deletion or the development of immune tolerance [57]. The low level of the cellular response contributed to a low level of protection during colonization of the intestinal enterocytes or muscles by larvae.

Neutrophil infiltration of the small intestinal epithelium has already been shown to contribute to the stimulation of epithelial cell cytokine production in rats infected with *T. spiralis*: The intestinal phase of the parasite infection influences the subsequent muscle invasion with increasing numbers of eosinophils and neutrophils [58]. Chitosan attracted neutrophils into the peritoneal cavity and reduced the local activation of the intestinal epithelium, thus possibly allowing more adult nematodes to survive [59]. Larvae in the muscle not exposed to the neutrophils attracted into the peritoneum, thus avoiding inflammation during nurse cell formation, would also be protected. This neutrophil accumulation may be caused by peritoneal cell damage provoked by highly adherent chitosan [46,60].

*T. spiralis* larvae express distinct carbohydrate moieties on antigens; these may possess immunogenic properties or help the parasite to evade the host immune response by various mechanisms [41]. The percentage of monocytes, B lymphocytes and other peritoneal cells expressing the CD23 receptor, a marker of allergy and helminth infection with a weak affinity for IgE, increased after infection but was slightly reduced by chitosan. The reduced CD23^+^ cell population would be associated with reduced immune activation [61] and weaker protection against intestinal stages of the nematode.

Chitosan treatment induced the production of significantly higher levels of IgA. The immunoglobulin recognized *T. spiralis* muscle larvae antigenic determinants rich in GlcNAc as WGA [62]. The IgA of mice injected with chitosan or infected with *T. spiralis* were seen to cross-react with the nematode antigens as a consequence of immunoglobulin affinity [63]: an increase in IgA antibody production may grow during *T. spiralis* infection [64,65]. It is important to stress that chitosan administration was also associated with an increased IgA response in the peritoneal cavity, together with an elevation in TGF-β, IL-10 and IL-6 production. TGF-β and IL-6 supported plasma cell survival and induced IgA secretion [66,67]. This increase in IgA production in response to chitosan units with GlcN residues is an interesting observation, as it illustrates the induction of a form of immune tolerance mechanism in response to a common residue in food, commensal bacterial components or parasites [68]. Recent studies have shown that GlcN possesses immunosuppressive activity, and being abundant in the molecules of the parasite, it may have immunomodulatory properties [53]. More detailed studies are needed to explain the immunoregulatory properties of separate residues released from biodegraded chitosan units.

In conclusion, high molecular weight chitosan administered into the peritoneal cavity of mice before nematode infection provoked an accumulation of myeloid cells in situ. After prolonged deposition and biodegradation of chitosan, immune activation was redirected into a tolerant milieu, resulting in a greater number of nematodes in the host.

## 4. Materials and Methods

### 4.1. Schedule of the Experiment

All experimental procedures were performed according to the Polish Law on Animal Experimentation and EU Directive 2010/63/UE. The study was approved by the First Warsaw Local Ethics Committee for Animal Experimentation (approval ID 151/2011).

Male seven-week-old C57BL6 mice were kept in standard light:dark conditions (12 h:12 h) with *ad libidum* access to water and pellet food. Experimental groups consisted of five mice.

High molecular weight chitosan with >75% degree of deacetylation was obtained from the chitin of crustacean shells (HMW; 310 to >375 kDa based on the viscosity range of 800–2000 mPaS) (Sigma, Steinheim, Germany). This was dissolved as 1% stock solution in 0.75% adipic acid (StanLab, Lublin, Poland) and autoclaved. The endotoxin level in stock solution was found to be <0.2 EU/mL in LAL assay (Pierce LAL chromogenic endotoxin quantitation kit; Thermo Fisher Scientific, Rockford, IL, USA). The animals were injected intraperitoneally with 200 μL amounts of 500 μg chitosan (final adipic acid concentration: 0.35%) daily for 10 days, starting five days before infection.

Attenuated Total Reflectance (ATR) spectra of the initial chitosan and peritoneal samples were recorded as described below.

*T. spiralis* larvae were isolated from BALB/c mice by artificial digestion of carcasses with pepsin/HCl solution. On day 5 after chitosan administration, the mice were alimentary infected with 400 larvae L1 *T. spiralis.* Four groups of mice were examined on days 5 and 30 after *T. spiralis* infection: mice infected with the nematode (5 DAI) and (30 DAI) but not treated with chitosan, infected mice previously treated with chitosan (Ch/5 DAI) and (Ch/30 DAI), mice which were uninfected and untreated (Ctr), mice only administered chitosan (Cht/Ctr). Adult forms were isolated from the small intestine using the Baermann technique. Muscle larvae were inspected after digestion of muscle tissue with pepsin/HCl solution. Ten females were cultured separately in RPMI 1640 Medium (Gibco, Paisley, UK) with 10% Glutamax (Gibco) for 48 h, and the number of newborn larvae (NBL) per female was estimated.

### 4.2. Cell Isolation, Cytokine and Myeloperoxidase Measurement

Cells were isolated from the peritoneal cavity; 5 mL of RPMI 1640 medium supplemented with penicillin/streptomycin (100 μg/mL) and 2 mM l-glutamine (all from Gibco), was injected into the peritoneal cavity and lavages were collected separately on ice and centrifuged (1200 rpm, 4 °C); the volume of collected fluid was measured. The supernatant was frozen at −80 °C for cytokine analysis. The cells were washed, counted with Trypan blue exclusion and a concentration of 2.5 × 10^6^ cells per mL was used for receptor phenotyping.

Peritoneal fluid was assayed for myeloperoxidase (MPO) and cytokines using commercially-available enzyme-linked immunosorbent assay (ELISA) reagents for MCP-1, TGF-β, IL-10 (e-Bioscences, San Diego, CA, USA) and TNF-α, IL-12p40, IL-4, IL-6 (BD Biosciences, Pharmingen, San Diego, CA, USA), MPO ELISA Kit (MyeloPeroxidase; R & D Systems, Minneapolis, MN, USA) according to the suppliers’ guidelines. For the TGF-β measurement, the samples were acidified and latent; samples were taken to measure the amount of active cytokine excreted into the culture medium. The plates were read at 490 nm or 450 nm (MPO) using a u-Quant spectrophotometer (Bio-Tek, Acton, MA, USA). The mean optical densities (OD) of the cultures, read in triplicate, were compared with the standard curves prepared using recombinant proteins.

### 4.3. Flow Cytometry

A suspension of 0.5 × 10^6^ cells was washed with cold PBS (phosphate buffered saline, pH 7.2, Gibco) and stained for 30 min at 4 °C with antibodies against surface markers coupled with flurochromes: CD11b-PercP or CD11b-APC, F4/80-PE, MHC class II-FITC, CD80-PE, CD86-APC, Dectin1-PercP Cy5, Mannose receptor (MR, CD206)-FITC, CD23-PE (AbD Serotec, Kidlington, UK) or IL-4Receptor-PE (BD Sciences, San Diego, CA, USA,). Unbound antibodies were washed out, cells were resuspended in 300 μL of PBS and analysed in a FACS Calibur cytometer with CellQuest and FCS Express 4 Flow (BD Sciences, San Jose, CA, USA) programs. Cells were gated into different populations based on F4/80 and CD11b expression. The level of cellular markers was presented as a percentage of positive cells in the CD11b^+^ population. The peritoneal suspension was smeared on a glass slide and stained with Giemsa for cell recognition.

### 4.4. Detection of IgA in the Peritoneal Fluid by ELISA

*T. spiralis* muscle stage larvae (L1), isolated from BALB/c mice 30 days post infection were prepared in PBS. The larvae were frozen three times and then sonicated in 0.1 M PBS, pH 7.4, using a VibraCell for six minutes (20 s on, 20 s off). Samples were centrifuged at 18,000× *g* for five minutes at 4 °C and the supernatant was stored at −80 °C until use. IgA in the peritoneal fluid was measured by ELISA; 96 well polysorp plates (Thermo Fisher Scientific, Nunc A/S, Roskilde, Denmark) were coated with 100 μL of 10 μg/mL *T. spiralis* L1 somatic extract or 10 μg/mL chitosan in a buffer pH 6.5 overnight in 4 °C. The wells were then blocked with 200 μL of 5% non-fat milk in PBS (pH 7.2) for one hour and incubated with 100 μL of undiluted peritoneal fluid for two hours at room temperature. RPMI medium was used as a negative control. After extensive washing with PBS Tween (0.05%) specific IgA was detected with anti-mouse IgA-HRP complex diluted 1:5000 (Novus Biologicals, Abingdon, UK); after a one hour incubation period, 100 μL TMB substrate (Sigma-Aldrich Inc., St. Louis, MO, USA) was added. The reaction was stopped after 20 min with 50 μL 2 N H_2_SO_4_ and absorbance was measured at λ = 450 nm with a μQuant spectrophometer (Bio-Tek, Acton, MA, USA).

### 4.5. IgA Specific Response Detected by Electrophoresis and Immunoblotting

Protein samples of *T. spiralis* L1 somatic extracts were boiled in Laemelli buffer and centrifuged for 10 min at 15,000× *g*; 10 μg of parasite antigen was separated on 4% and 12% SDS PAGE gel for 50 min at a constant 150 V using a Bio-Rad TetraCell system (Bio-Rad Laboratories, Richmond, BC, Canada). The proteins were transferred onto a nitrocellulose membrane (Sigma, 0.45 μm pore size) for one hour at 18 V with SemiDry Transfer apparatus (BioRad Laboratories, Hercules, CA, USA). After transfer, the membrane was blocked with 5% non-fat milk in PBS (pH 7.2) for two hours. Subsequently, membranes were incubated overnight in 4 °C with undiluted peritoneal fluid. After extensive washing with PBS Tween (0.05%), the membrane was incubated with anti-mouse IgA-HRP complex diluted 1:5000 (Novus Biologicals) for one hour. Protein bands were detected with DAB/H_2_O_2_ solution for 20 min. The membrane was dried and analyzed in a Molecular Imager Gel Doc^TM^ XR^+^ visualization system.

### 4.6. Identification of N-Acetyl-glucosamine Residues in T. spiralis Somatic Antigen

After electrophoresis and transfer of proteins onto nitrocellulose, the membranes were blocked with 1% BSA in PBS. *N*-acetyl-glucosamine epitopes on *T. spiralis* antigens were developed using a Biotinylated Lectin Kit I (Vector Laboratories, Burlingame, CA, USA) using Wheat Germ Agglutinin (WGA). The blots were washed with 10 mM TRIS-buffered saline (TBS, pH 7.4), blocked overnight with 3% (*w*/*v*) bovine serum albumin (Sigma) in TBS (TBS-BSA), washed again with TBS and incubated for two hours with lectin diluted in TBS-BSA 1:1000. After extensive washing with TBS, the blots were incubated for two hours at room temperature in TBS-BSA containing avidin-alkaline phosphatase (Sigma-Aldrich Inc., St. Louis, MO, USA) diluted 1:100,000. The blots were washed with TBS and subsequently developed using BCIP/NBT (SIGMA FAST™ 5-bromo-4-chloro-3-indolyl phosphate/nitro blue tetrazolium. Sigma-Aldrich Inc.). The reaction was stopped by bathing the blots in distilled water.

### 4.7. Fourier Transform Infrared Spectroscopy (FTIR) Analysis

Samples of 200 μL of peritoneal fluid were taken from mice injected with 5 mL of RPMI at 37 °C after a 1.5 min massage of the abdomen. Samples were evaluated after being cooled to 4 °C, before being frozen at −20 °C until use. Attenuated Total Reflectance (ATR) analyses of the initial chitosan and peritoneal samples were recorded on a Genesis II FTIR spectrophotometer (Mattson, Madison, WI, USA) equipped with an ATR device (MIRacleTM PIKE Technologies, Madison, WI, USA) and a zinc selenide (ZnSe) crystal. For FTIR analysis, the initial chitosan and peritoneal samples were poured on the spectrophotometric windows from CaBr_2_ (Sigma-Aldrich) and evaporated at room temperature (25 °C) for 24 h. FTIR spectra were collected in the wavenumber range between 4000 cm^−1^ and 600 cm^−1^, at a resolution of 4 cm^−1^ using 64-times scanning [69].

### 4.8. Histopathological Examination of Granuloma on the Surface of the Liver

The organs were isolated and immediately immersed in Neg-50™ Frozen Section Medium (Thermo Scientific, Dreieich, Germany) and frozen in liquid nitrogen. The samples were stored at −80 °C and then cut into 10 μm sections in cryomicrotome at −20 °C, and fixed at 90% methanol for five minutes. Standard staining with Cole’s hematoxylin and acid eosin Y was performed. The preparations were observed under a light microscope.

### 4.9. Statistical Analysis

The results are presented as mean and standard error. Data was analyzed with ANOVA and the Student’s *t*-test. Differences between groups were considered significant when the *p* value < 0.05.

## 5. Conclusions

Chitosan appears to augment cellular infiltration, with an immunoregulative phenotype supporting *T. spiralis* infection, and its biodegraded particles, rich in *N*-acetyl-d-glucosamine and d-glucosamine, may be involved in the process. These findings may be relevant for both people and animals treated with chitosan preparations.
